# An Exponentiation Method for XML Element Retrieval

**DOI:** 10.1155/2014/404518

**Published:** 2014-02-13

**Authors:** Tanakorn Wichaiwong

**Affiliations:** Department of Computer Science, Faculty of Science, Kasetsart University, Bangkok 10903, Thailand

## Abstract

XML document is now widely used for modelling and storing structured documents. The structure is very rich and carries important information about contents and their relationships, for example, e-Commerce. XML data-centric collections require query terms allowing users to specify constraints on the document structure; mapping structure queries and assigning the weight are significant for the set of possibly relevant documents with respect to structural conditions. In this paper, we present an extension to the MEXIR search system that supports the combination of structural and content queries in the form of content-and-structure queries, which we call the Exponentiation function. It has been shown the structural information improve the effectiveness of the search system up to 52.60% over the baseline BM25 at MAP.

## 1. Introduction

Nowadays, the XML (http://www.w3.org/TR/xml11/) research is willing increasingly more documents having the structure with respect to certain structural [1]. Exploiting this structure is a significant part of improving retrieval effectiveness which can be divided into two categories: using document structure and user queries. Several form of the document's structure based retrieval models have been developed, such as BM25F [2] ranking function that is composed of several document fields with potentially different degrees of importance; PRM-S [3] is based on probabilistic retrieval model; and FRM [4] is the relevance feedback function based on the language model. Broschart and Schenkel presented the proximity weighting to improve the search system [5]. On the other hand, it is based on user queries, such as QRX [6] which is based on tree matching model without knowing the exact structure of the data, using the similarity measure of the vector space model. Unfortunately, this method has a drawback on the efficiency issue. The weight has been based on depth of the path and location in the document logical structure and then used as probabilities function based on the language model [7]; the length has been used as a normalization incorporated through a prior probability in the ranking function [8]. In [9, 10], highlight the structure weight in TopX (http://topx.sourceforge.net/) search engine. It assigns a small constant and tunable score for every navigational condition that is matched to query by using the frequency of the tag name. The weight has also been calculated based on the distribution of tag names which is used in a way similar to the binary independence retrieval model, but investigating the presence of tags in relevant and nonrelevant elements, to estimate the tag weights [11]. In [12], it is shown the structure does not improve the effectiveness of the retrieval system much because the users are very bad at giving structural hints with respect to INEX-IEEE collection and it requires further investigation. In this paper, we are investigating retrieval technique and related issues over a strongly structured collection of XML documents with the Initiative for the Evaluation of XML Retrieval (INEX) (https://inex.mmci.uni-saarland.de/) collections based on user queries. With richly structured XML data, we have been shown that the structural information using the Exponentiation function could be utilized to improve the effectiveness of search systems.

This paper is organized as follows.  Section 2 reviews the data model and notions.  Section 3 explains the presents state of the art approaches.  Section 4 shows the experiment results and discussion; conclusions and further work are drawn in Section 5.

## 2. Data Model and Notions

In this section, we provide some historical perspectives on areas of XML research that have influenced this article as follows.

### 2.1. XML Indexing Methods

**Figure 1:**
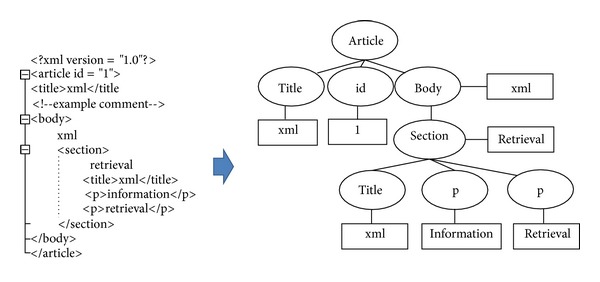
The Example of XML Element Tree.

The basic XML data model is a labeled, ordered tree.  Figure 1 shows the data tree of an XML document based on the node-labeled model.

**Figure 2:**
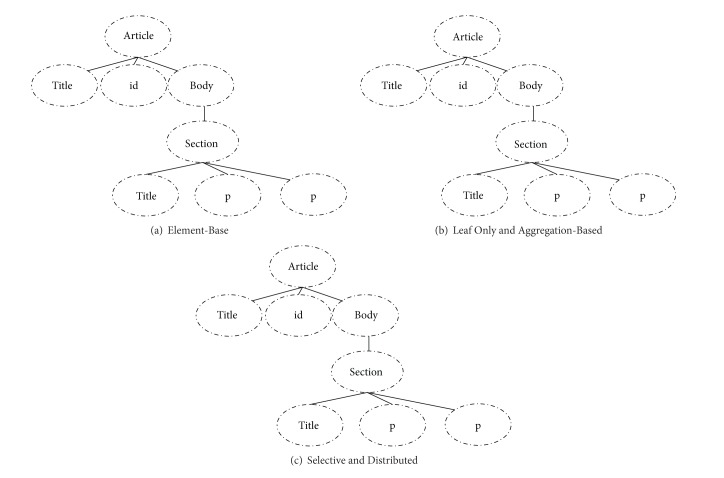
Illustrations of some of the indexing strategies.

Classical retrieval models have been adapted to XML retrieval. Several indexing strategies have been developed in XML retrieval as shown in Figure 2.

Element Base indexing [8] allows each element to be indexed on the basis of both direct text and the text of descendants. This strategy has a major drawback in that it is highly redundant. Text occurring at the *n*th level of the XML logical structure is indexed n times and thus requires more index space. This strategy is illustrated in Figure 2(a), where all elements are indexed. Leaf-Only indexing [13] allows indexing of only leaves through element or elements directly related to text. This strategy addresses the redundancy issues noted above. However, the propagation algorithm for the retrieval of nonleaf elements requires a certain level of efficiency. This strategy is illustrated in Figure 2(b), where the leaf elements are indexed. Aggregation-Based indexing [14] uses the concatenated text of an element to estimate a term statistic. This strategy has been used to aggregate term statistics directly on the basis of the text and its descendants. This is illustrated in Figure 2(b), where the leaf elements are indexed. Selective indexing [13, 15] involves eliminating small elements and elements of a selected type; this strategy is illustrated in Figure 2(c), where only semantic elements are indexed. Distributed indexing [15] is separately created for each type of element in conjunction with the selective indexing strategy, as shown in Figure 2(c). The ranking model runs each index separately and retrieves ranked lists of elements. These lists are merged to provide a single rank across all element types. To merge lists, normalization is performed to take into account the variation in elements size across the different indices such that scores across indices are comparable.

### 2.2. XML Query Languages

Querying in structured documents must be with respect to content and structure. INEX identified two types of queries [23, 24]; they are content only (CO) and content and structure (CAS) as follows.

#### 2.2.1. Content Only Queries

These queries are formed by ignoring the document structure, in the same way as the traditional queries used in IR collections. However, they pose a challenge to XML retrieval in that the retrieval results in returning document components, that is, XML elements instead of whole documents in response to a user query. Queries can be elements of various complexities, that is, at different levels of the XML document's structure. This is suitable for XML retrieval where users do not know or are not concerned about the structure, that is, with the logical organization of the document, when expressing their information needs. For example, the best answer for a query “XML retrieval” applied to Figure 1 may be a “section” and not “title” or “p” elements.

#### 2.2.2. Content-and-Structure Queries

These queries contain conditions of both content and structure. These conditions may refer to the content of specific elements and specify the type of requested answer elements. However, the complexity and the expressiveness of content-and-structure query languages are difficult for the end users because they have to know the logical organization of the document when expressing their information needs. Trotman and Lalmas [12] showed that the structure did not improve the effectiveness of the retrieval system very much because users were normally not capable of giving useful structural hints with respect to INEX-IEEE collection. However, the content-and-structure query can be very useful for expert users in specialized scenarios.

#### 2.2.3. The Narrowed Extended XPath I

The Narrowed Extended XPath I (NEXI) query language was developed at INEX [25] as a simple query language for content-oriented XML retrieval evaluation. The enhancement comes from the introduction of a new function named “about()”. The “contains()” function of XPath, which requires an element (its text) to contain the given string content, was replaced by the “about()” function, which requires an element to be about the content. The NEXI query provides support for the descendant axis as follows. //*T*[*t*] is simple elements with paths matching *T* and contents about *t*. //*S*[*s*]//*T* returns elements *T* which are descendants of the element *S*, where the element *S* contains *s*. //*S*[*s*]//*T*[*t*] returns elements *T* which are descendants of the element *S*, where the element *S* contains *s* and the element *T* contains *t*.

### 2.3. Structure Weight IR

Schlieder and Meuss presented the QRX [6] which is based on tree matching without knowing the exact structure of the data of the similarity measure of the vector space model; an element score is computed as follows:

(1) $$\begin{array}{c} \text{Score}\left(e,q\right)=\underset{t\in q}{\sum }tf_{t}*idf_{t}. \end{array}$$

Stephen et al. [2] and Robertson and Zaragoza [26] present BM25F as an extension of the baseline BM25 [27] scoring function that is adapted to score field documents. Using the BM25F scheme presented in [28], an element score is computed as follows:

(2) $$\begin{array}{c} \text{Score}\left(e,q\right)=\underset{t\in q\cup e}{\sum }\frac{tf_{e,t}}{K+tf_{e,t}}*W_{t}, \end{array}$$

where Score(*e*, *q*) measures the relevance of element *e* to query *q*, *tf*_*e*,*f*_ is a weighted normalized term frequency,  *K* is a common tuning parameter for the BM25, and *W*_*t*_ is the inverse document frequency weight of term *t*.

The weighted normalized term frequency is obtained by first performing length normalization on term frequency *W*_*e*,*f*,*t*_ of term *t* in field *f* in element *e* as follows:

(3) $$\begin{array}{c} W_{e,f,t}=\frac{tf_{e,f,t}}{1+B_{f}*\langle \left(\text{le}{\text{n}}_{e,f}/\text{avgle}{\text{n}}_{f}\right)-1\rangle }, \end{array}$$

where *B*_*f*_ is a smoothing parameter,  len_*e*,*f*_ is the length of field *f*, and avglen_*f*_ is the average length of elements in the entire collection after multiplying the normalized term frequency *W*_*e*,*f*,*t*_ by field weight *W*_*f*_:

(4) $$\begin{array}{c} tf_{e,t}=\underset{f}{\sum }W_{f}*W_{e,f,t}. \end{array}$$

Kim and Croft [4] recently introduced the Field Relevance Model (FRM). FRM employs the notion of field relevance and a corresponding retrieval model between query terms and document fields, which are calculated by *Field Relevance* given a query *q* = *q*_1_,…, *q*_*m*_, and *field relevance* *P*(*F*_*j*_ | *q*_*i*_, *R*) is the distribution of per-term relevance over document fields. *Field Relevance Model* is based on field relevance estimates *P*(*F*_*j*_ | *q*_*i*_, *R*); the *Field Relevance Model* combines field-level scores *P*(*q*_*i*_ | *F*_*j*_, *D*) for each document using field relevance instead of weights as follows:

(5) Score(e,q)=∏1≤i≤m ∑1≤j≤nλP(Fj ∣ qi,R)+(1−λ)P(qi ∣ Fj,D).

Broschart and Schenkel [5] presented the use of proximity-aware scoring functions that lead to significant effectiveness improvements for XML retrieval. This method introduces modified proximity scores that take the document structure as follows:

(6) $$\begin{array}{c} \text{Score}\left(e,q\right)=W_{t,e}+\text{Pro}{\text{x}}_{t,e},\\ W_{t,e}=\underset{t\in q}{\sum }\frac{\left(k_{1}+1\right)*tf_{t}}{K+tf_{t}}*ief_{t},\\ ief_{t}=\log\left[\frac{N-e_{t}+0.5}{e_{t}+1}\right]. \end{array}$$

To compute the proximity part of the score for each term *t*, at first compute an accumulated score acc_*t*_ that depends on the distance of this term's occurrences in the element to other terms, adjacent query term occurrences using for each adjacent occurrence of a term *t*_*j*_ at distance *d* to an occurrence of *t*_*i*_, the acc_*t*,*i*_ grows by (*ief*_*t*_)/*d*. The proximity score is computed as follows:

(7) $$\begin{array}{c} \text{Pro}{\text{x}}_{t,e}=\underset{t\in q}{\sum }\min\left\{1,ief_{t}\right\}\frac{\left(k_{1}+1\right)*\text{ac}{\text{c}}_{t}}{K+\text{ac}{\text{c}}_{t}}, \end{array}$$

where Score(*e*, *q*) measures the relevance of element *e* to a query *q*, acct_*t*_ is calculated by (*ief*_*t*_)/*d*.

Ogilvie and Callan [7] is based on language models and employs element-based indexing. Given a query *q*, terms *t*_*i*_ for each element *e* and its corresponding element language model ⊖_*e*_, the element *e* is ranked as follows:

(8) $$\begin{array}{c} P\left(e\;|\;q\right)=P\left(e\right)*P\left(q\;|\;{⊖}_{e}\right), \end{array}$$

where  *P*(*e*) is the probability of relevance for element *e* and *P*(*q* | ⊖_*e*_) is the probability of the query *q* generated by language model ⊖_*e*_. For instance,

(9) $$\begin{array}{c} P\left(t_{1},\ldots ,t_{n}\;|\;{⊖}_{e}\right)=\overset{n}{\underset{i=1}{\prod }}\lambda P\left(t_{i}\;|\;e\right)+\left(1-\lambda \right)P\left(t_{i}\;|\;C\right), \end{array}$$

where  *P*(*t*_*i*_ | *e*) is estimation of term *t*_*i*_ in element *e*, *P*(*t*_*i*_ | *C*) is the probability of term *t*_*i*_ in collection *C*, and *λ* is the smoothing parameter.

To account for the length of an element *e*, and in particular for the heavily biased distribution of small elements in XML documents, which can be used to set *P*(*e*) as follows [8]:

(10) $$\begin{array}{c} P\left(e\right)=\frac{\text{lengt}{\text{h}}_{e}}{\sum_{C}^{}\text{lengt}{\text{h}}_{e}}, \end{array}$$

where length_*e*_  is the length of element *e* and ∑_*C*_length_*e*_ is the length of element *e* occurring in collection *C*.

Theobald et al. [10] present the extended BM25 function in the TOPX, which is known as the Compactness of the baseline BM25 as follows:

(11) Score(e,q) =∑t∈q∪e(k1+1)∗tft,ek1∗((1−b)+b∗(len(eA)/avelA))+tft,e  ∗log⁡〈(NA−et+0.5)/et〉  NA+0.5,

where len(*e*_*A*_) is the length of element *e* with tag *A*, avel_*A*_ is the average length of elements in the entire collection with tag *A*, *k*_1_, and *b* is a common tuning parameter for the BM25.

The modified function provides a dampened influence of the *tf*_*t*,*e*_ with tag *A*. However, this strategy is limited in that each tag name must be the same to implement automatic grouping and weight calculation.

The idea is to associate a weight to a structural constraint to reflect its significance. These weights are then used in the scoring function used to estimate an element relevance.With the increased availability of the data-centric a need for query in both structure and content of the XML documents has become explicit. As a result, a more complex information source is available, in fact, allowing us to improve the performance of search systems. Our approach considers the use of structure weight method, as discussed in Section 3.

## 3. Method

In this section, the search results become more refined at every step, and the refinement ultimately narrows down a set of potentially interesting documents. Below we describe our approach in more details.

### 3.1. Step  1: Elements Score

Firstly, we defined Score(*e*, *A* = *t*) is a score for the relevance of a term *t* of an element *e* and then we used the baseline BM25 [27] in Sphinx (http://sphinxsearch.com/) [29] formula to score the element nodes according to query terms *t* contained in content conditions as follows:

(12) Score(e,A=t)  =Wt∗(k1+1)∗tft,ek1∗〈(1−b)+b∗(len(e)/avel)+tft,e〉,

where Score(*e*, *A* = *t*) measures the relevance of element *e* to query term *t*, *tf*_*t*,*e*_ is the frequency of term *t* occurring in element *e*, len(*e*) is the length of element *e*, avel is the average length of elements in the entire collection, and *k*_1_ and *b* are used to balance the weight of term frequency and element length.

And then, we compute the inverse element frequency *W*_*t*_ as follows:

(13) $$\begin{array}{c} W_{t}=\frac{\log\langle \left(N-e_{t}+1\right)/e_{t}\rangle }{\log\left(N+1\right)}, \end{array}$$

where *W*_*t*_ is the inverse element frequency weight of term *t*, *N* is the total number of an element in the entire collection, and *e*_*t*_ is the total element of a term *t*occur.

For an “about()” function in NEXI operator with multiple terms that appeared to an element *e*, the aggregated score of *e* is simply computed as the sum of the element's scores for each term *t*_1_,…, *t*_*n*_ conditions as follows:

(14) Score(e,q)=Score(e,A[about(t1,…,tn)])=∑ti∈nScore(e,A=ti).

### 3.2. Step  2: Score Sharing Function

In the second step of our approach [30], we compute the scores of all elements from (14), in the collection that contains query terms. We consider the scores of elements *e* by accounting for their relevant descendants *e*_*c*_. The scores of retrieved elements Score(*e*, *q*) are now shared between the leaf node and their parents in the document XML tree according to the following scheme:

(15) $$\begin{array}{c} \text{Score}\left(e,q\right)⟵\text{Score}\left(e,q\right)+\langle \overset{}{\underset{e,c}{\sum }}\text{Score}\left(e_{c},q\right)*\beta^{n}\rangle , \end{array}$$

where Score(*e*, *q*) is a current parent node, Score(*e*_*c*_, *q*) is a relevant child of element *e*, and *β* is a tuning parameter.

 
*IF *{0 − 1}  *THEN* preference is given to the leaf node over the parents.
 
*OTHERWISE*, preference is given to the parents.
 
*n* is the distance between the current parent node and the leaf node.

### 3.3. Step  3: Exponentiation Weight Function

The third step of our approach is the structure score evaluation. To improve the search result with richly structured, we assume that a query is composed of content (keywords) and structure constraints. The document-query similarity is evaluated by considering content and structure separately. We then combine these scores to the set of possibly relevant elements. Our structural scoring model essentially counts the number of navigational (i.e., element name-only) query conditions that are satisfied by a result candidate and thus considering the content conditions matched for the user queries. It assigns *c*_*e*_ for every directional condition that matched the element name *e*_name_ ∈ *d*_path_ (i.e., an absolute path on the document structure). We analysed the structure for each topic in INEX as shown in Table 1 with respect to the INEX content-and-structure queries and each topic is including a few structure indications. Thus, we are proposed the novel of structural scoring when the user query is matching the structural constraints against the document tree using the *Exponentiation* is *a* ^*n*^.

**Figure 3:**
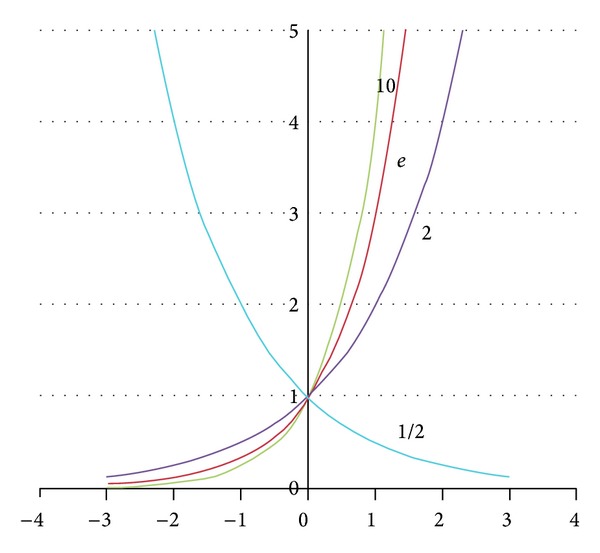
Variation in the value of base *a* ^*n*^ parameter.

In order to evaluate the sensitivity of the *Exponentiation*, we have variation in the value of *a* parameter, including *base 10*, *base e*, *base 2*, *and base 1/2* as shown in Figure 3. According to the trend of the graph more smooth than other values and the powers of 2 are important in computer science because there are 2^*n*^ possible values for an *n*-bit binary variable. Thus, we simply for our algorithm calculate base on 2^*c*_*e*_^. After that we recomputed the element score Score(*e*, *q*) as follows:

(16) $$\begin{array}{c} c_{e}=\underset{e_{\text{name}}\in d_{\text{path}}}{\sum }e_{\text{name}},\\ \text{Score}\left(e,q\right)⟵\text{Score}\left(e,q\right)*\langle 2^{c_{e}}\rangle , \end{array}$$

where *c*_*e*_ is the frequency of navigational condition that is matched with the *e*_name_ ∈ *d*_path_.

In the following, we define *T*(*d*) as the set of all elements in *d* that match the target element of the query. In document mode, every document *d* inherits the aggregated score among all target elements *e*, and these document scores Score(*d*, *q*) determine the output ranking among documents as follows:

(17) $$\begin{array}{c} \text{Score}\left(d,q\right)=\underset{e\in T\left(d\right)}{\sum }\text{Score}\left(e,q\right). \end{array}$$

To see how users use structure in their queries, for instance, the user query needs “retrieve document sections with the paragraph *p* contains *xml retrieval*” as follows:

 //section[about(//p, “xml retrieval”)]

**Figure 4:**
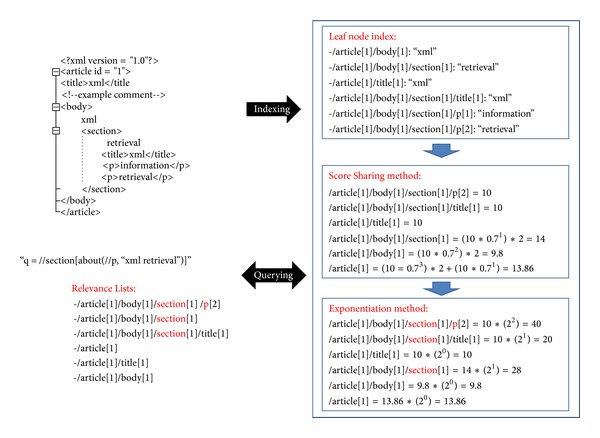
An Example of Exponentiation processing.

The first filter looks for occurrences of the term “xml” and “retrieval” in elements *e* whose context matches the path “//section//p” on the *d*_path_. It is possible to assigning more weight for the return element *e*. In this case, we assume the Score(*e*, *q*) for each element *e* is 10, *β* is 0.7 and then the calculations are shown in Figure 4.

Thus, the Score(*d*, *q*) for the document *d* is 〈40 + 20 + 10 + 28 + 9.8 + 13.86〉 = 121.66.

## 4. Experiment Setup

In this section, we present and discuss the results based on the INEX collection. This experiment was performed on Intel Pentium i5 4 ∗ 2.79 GHz with 6 GB of memory, Microsoft Windows 7 Ultimate 64 bit Operating System and Microsoft Visual C*♯*.NET 2008.

### 4.1. INEX Collection

The INEX-IMDB collection used in INEX 2010 (https://inex.mmci.uni-saarland.de/) was generated from the plain text files published on the IMDB web site on April 10, 2010. There are two kinds of objects in the collection, movies and persons involved in movies. Each object is richly structured. For example, each movie has title, rating, directors, actors, and so forth; each person has name, birth date, and so forth. In total, the IMDB data collection contains 4,418,081 XML documents, including 1,594,513 movies, 1,872,471 actors, 129,137 directors who did not act in any movie, 178,117 producers who did not direct or act in any movie, and 643,843 other people involved in movies who did not produce or direct or act in any movie.

### 4.2. INEX Evaluations

The effectiveness of the retrieval results will be evaluated using the metrics as that in traditional IR, for example, precision, recall, MAP, P@10, P@20, and P@30 [31, 32]. Given a topic *T* and a set of documents *D*, each tested IR system returns an ordered subset *S* = *s*_1_,…, *s*_*n*_ of *D*, ranked by the system's estimate of the likelihood that each document is relevant to *T*. Several effectiveness measures are computed, including average precision (AP); precision at *k* returned documents (P@*k*) defined as follows:

(18) $$\begin{array}{c} \text{AP}=\frac{\sum_{k=1}^{\left|S\right|}\mathrm{rel}\left(s_{k}\right)*\text{P}@k}{R},\\ \text{P}@k=\frac{\sum_{i=1}^{k}\mathrm{rel}\left(s_{i}\right)}{k},\\ R=\underset{d_{i}\in D}{\sum }\mathrm{rel}\left(d\right). \end{array}$$

Performance across a set of topics is measured by calculating the mean of the AP values obtained by the measure for each individual topic, resulting in MAP. Assuming there are *n* topics:

(19) $$\begin{array}{c} \text{MAP}=\frac{1}{n}*\overset{n}{\underset{t=1}{\sum }}\text{A}{\text{P}}_{t}. \end{array}$$

### 4.3. Results and Discussion

In this section, we tuned the *β* parameter using INEX-2005 ad hoc track evaluation scripts distributed by the INEX organizers. Our tuning approach was such that the sums of all relevance scores are maximized and then the total number of leaf node is 2500 and the *β* parameter is set to 0.60. Following that, we used the Sphinx parameters for the BM25 where *k*_1_ = 1.20 and *b* = 0.00 and the entire Sphinx match mode values in our experiment include MATCH ANY (TF), MATCH PHRASE (PHRASE), and MATCH EXTENDED (BM25) and are provided in Table 2. The main components of the MEXIR [33] retrieval system are as follows.

1. When new documents are entered into the system, the Absolute Document XPath Indexing (ADXPI) [34] indexer parses and analyzes the name of each element and its position to build inverted lists for each index in this system.
2. The SphinxDB search engine is used to build both indices in the system. The Selected Weight index is based on term frequency, and the Leaf Node index is based on the classic BM25 function.
3. The Score Sharing function is used to assign parent scores by assigning a proportion of the scores of the leaf nodes to their parents using a top-down approach.
4. The Exponentiation function is used to adjust the element scores based on linear combination.

The MEXIR search engine retrieves XML elements based on the leaf node indexed with respect to the significant words including the Exponentiation and Score Sharing functions, and then we combine relevance score from the element into the document score. Thus, the document with the higher relevance score will be chosen as the retrieval set. The details of experiment are shown in Table 3.

The performance of different features and ranking methods can now be evaluated. In order to deepen into the analysis of the Exponentiation scoring function, we have also run experiments to study the impact of structure weight with the content-and-structure query in the performance.  Table 4 shows the results compared for the best performing runs with and without Exponentiation technique. The **p16-BM25-EXPO** used the Exponentiation for boosting element score, and the **p16-BM25** is the baseline BM25 and then the Exponentiation function was shown to improve the effectiveness of search system measured in terms of MAP, P@10, P@20, and P@30 and are 52.60%, 50.60%, 54.16%, and 58.79%, respectively.  Table 5 shows the results compared for the best performing runs with and without the Score Sharing technique. The **p16-BM25-EXPO** is used the Exponentiation and the used the Score Sharing is the **p16-SS-SW** and then the Exponentiation weight shown improve the effectiveness of over the Score Sharing technique measured in terms of MAP, P@10, P@20 and P@30 are 81.58%, 82.92%, 75.09% and 67.83%, respectively. It can be seen, that **p16-BM25-EXPO** obtained the best performance, although the improvement over both the baseline BM25 and the Score Sharing is significant for most of the considered metrics. The significance (*P*) was computed with a 2-tailed *t*-test as shown in Table 6. The **p16-BM25-EXPO** improved by 0.48% over the baseline BM25 at MAP, and 0.75% over the baseline BM25 with the Score Sharing at MAP on INEX-IMDB collection.

In this analysis, we take the results that were obtained from BM25 over the Exponentiation and compare them with the results from the baseline BM25 and over the Score Sharing function. It is shown again that Exponentiation works well with the document-centric XML documents. We can conclude that significant improvement of results of the Exponentiation function can be obtained from the content-and-structure query and document structure. This finding suggests that it is possible to improve the TF, PHRASE, and the baseline BM25 approaches, which are the usual benchmarks in INEX. The main conclusion that can be drawn from the experiments is that the Exponentiation function is successful in structure weight and could be utilized to improve the effectiveness of search systems.

Another major conclusion, is that we analyzed the effectiveness of the runs for each of the three topic types with respect to the INEX [17] and the results are presented in Tables 7, 8, and 9. The overall results are satisfactory if we compare them with those obtained by participants in the INEX contests. On comparing the effectiveness for the informational topics, our run ranked first, scoring 0.3564, measured with MAP; it ranked fifth scoring 0.6667, measured with 1/Rank for the known-item topics; and in the results of the list topics, our run ranked first, scoring 0.4251, measured with MAP.

In this analysis, we take the results that were obtained from the INEX report [17]. It is shown again that our system works well with the *List* and *Informational* topics of the document-centric XML documents measured with the MAP metric. Unfortunately, on the known-item topics, the relevant answer is a single document; in this area, the performance was not satisfactory and so further investigation is required.

## 5. Conclusions

With the increased availability of the data-centric a need for query in both structure and content of the XML documents has become explicit. As a result, a more complex information source is available, in fact, allowing us to improve the performance of search systems. In this paper, we are investigating retrieval techniques and related issues over a strongly structured collection using the Exponentiation weight for the document's structure over the content-and-structure query, in the data-centric track of the INEX 2011. Our expectation is that structure weighted will improve the effectiveness of the search systems. In terms of processing time, our system required an average of one second per topic. In addition, our run for the ad hoc task showed that the structural information could be utilized to improve the effectiveness of the search system over the baseline BM25 measured in terms of MAP, P@10, P@20, and P@30 and are 52.60%, 50.60%, 54.16%, and 58.79% and over the Score Sharing technique measured in terms of MAP, P@10, P@20, and P@30 and are 81.58%, 82.92%, 75.09%, and 67.83%, respectively. The success of our ad hoc run indicates that indexing the complete XML structure of IMDB and the structure weights are necessary for effective document retrieval in the search system.

In future work, we will look closer at the relative value of various types of metadata, tags, and subject headings. We will also look at the different weighting methods underlying the relevance judgements and topic categories, such as blind feedback and recommendation search.

## Figures and Tables

**Table 1:** Report for structure in CAS topics of INEX.

CAS topics	Max.	Min.	Avg.
INEX-2006	5	1	**2.65**
INEX-2007	6	1	**2.87**
INEX-2008	4	1	**2.68**
INEX-2009	5	1	**2.47**
INEX-2010	4	1	**2.57**
INEX-2011	11	1	**2.75**

The bold font refer to the % that use to calculate value of improvement.

**Table 2:** The sphinx search modes.

Mode	Description
Match any	The final weight is a sum of weighted phrase ranks for matching *any* of the query words.
Match phrase	The final weight is the sum of weighted phrase ranks for matching the query *phrase*, which requires a perfect match.
Match extended	The final weight is the sum of weighted phrase ranks and the *BM25* weight, multiplied by a thousand and rounded to the nearest integer.

**Table 3:** The details of experiments.

Run	Exponentiation	Score Sharing
p16-BM25-EXPO	Yes	No
p16-TF-EXPO	Yes	No
p16-PHRASE-EXPO	Yes	No
p16-BM25	No	No
p16-TF	No	No
p16-PHRASE	No	No
p16-BM25-SS	No	Yes
p16-TF-SS	No	Yes
p16-PHRASE-SS	No	Yes

**Table 4:** Compare performing runs based on MAP with and without the exponentiation.

Run	MAP	P@10	P@20	P@30
**p16-BM25-EXPO**	0.3479	0.4316	0.3645	0.3298
p16-TF-EXPO	0.2125	0.2500	0.2171	0.1930
p16-PHRASE-EXPO	0.1937	0.2342	0.1921	0.1675
**p16-BM25**	0.1830	0.2184	0.1974	0.1939
p16-TF	0.0857	0.1447	0.1118	0.0921
p16-PHRASE	0.0857	0.1447	0.1118	0.0921
**%**	**52.60**	**50.60**	**54.16**	**58.79**

The bold font refer to the % that use to calculate value of improvement.

**Table 5:** Compare performing runs based on MAP with and without the score sharing.

Run	MAP	P@10	P@20	P@30
**p16-BM25-EXPO**	0.3479	0.4316	0.3645	0.3298
p16-TF-EXPO	0.2125	0.2500	0.2171	0.1930
p16-PHRASE-EXPO	0.1937	0.2342	0.1921	0.1675
**p16-BM25-SS**	0.0641	0.0737	0.0908	0.1061
p16-TF-SS	0.0641	0.0711	0.0882	0.1044
p16-PHRASE-SS	0.0606	0.0605	0.0829	0.1070
**%**	**81.58**	**82.92**	**75.09**	**67.83**

The bold font refer to the % that use to calculate value of improvement.

**Table 6:** The significance (*P*) is computed with a 2-tailed *t*-test at MAP.

	Run	MAP
BM25	p16-BM25-EXPO	0.3479
	p16-BM25	0.1830
	*P* (*t*-test)	**0.48**
Score Sharing	p16-BM25-EXPO	0.3479
	p16-BM25-SS	0.0641
	*P* (*t*-test)	**0.75**

The bold font refer to the % that use to calculate value of improvement.

**Table 7:** Best performing runs based on MAP over the information topics.

Run	MAP	1/Rank	P@10	P@20
**p16-BM25-EXPO **[16]	**0.3564**	**0.8000**	**0.5000**	**0.4200**
p30-2011CUTxRun2 [17]	0.3449	0.7067	0.5000	0.4700
p47-FCC-BUAP-R1 [18]	0.3219	1.0000	0.5600	0.4300
p2-ruc11AMS [19]	0.3189	0.6500	0.4200	0.4500
p4-UAms2011adhoc [20]	0.3079	0.6750	0.3800	0.3100
p18-UPFbaseCO2i015 [21]	0.2576	0.6346	0.4600	0.4400
p77-PKUSIGMA02CLOUD [17]	0.2118	0.5015	0.4400	0.4200
p48-MPII-TOPX-20-co [17]	0.0900	0.3890	0.2600	0.1800
p12-IRIT-focus-mergeddtd-04 [22]	0.0366	0.3022	0.2200	0.1100

The bold font refer to the % that use to calculate value of improvement.

**Table 8:** Best performing runs based on 1/Rank over the known-item topics.

Run	MAP	1/Rank	P@10	P@20
p4-UAms2011adhoc	0.8112	0.9167	0.3167	0.2417
p2-ruc11AS2	0.7264	0.9167	0.3167	0.2417
p48-MPII-TOPX-20-co	0.2916	0.7222	0.2333	0.1833
p18-UPFbaseCO2i015	0.3752	0.7104	0.2500	0.2083
**p16-BM25-EXPO**	**0.4745 **	**0.6667**	**0.0833**	**0.0417**
p77-PKUSIGMA01CLOUD	0.5492	0.6389	0.3167	0.2417
p30-2011CUTxRun2	0.3100	0.5730	0.2667	0.1750
p47-FCC-BUAP-R1	0.2500	0.3333	0.0333	0.0167
p12-IRIT-large-nodtd-06	0.0221	0.0487	0.0167	0.0333

The bold font refer to the % that use to calculate value of improvement.

**Table 9:** Best performing runs based on MAP over the list topics.

Run	MAP	1/Rank	P@10	P@20
**p16-BM25-EXPO**	**0.4251**	**0.7778**	**0.4778**	**0.3833**
p4-UAms2011adhoc	0.3454	0.6674	0.4222	0.3500
p77-PKUSIGMA02CLOUD	0.3332	0.5432	0.3889	0.3667
p2-ruc11AS2	0.3264	0.6488	0.4111	0.3333
p48-MPII-TOPX-20-co	0.2578	0.4926	0.3000	0.3333
p18-UPFbaseCO2i015	0.2242	0.5756	0.3556	0.3278
p12-IRIT-focus-mergeddtd-04	0.1532	0.2542	0.2333	0.2111
p30-2011CUTxRun3	0.0847	0.5027	0.1889	0.1611
p47-FCC-BUAP-R1	0.0798	0.3902	0.2889	0.2500

The bold font refer to the % that use to calculate value of improvement.

## References

[1] Kamps J, Marx M, de Rijke M, Sigurbjornsson B Structured queries in xml retrieval.

[2] Stephen R, Hugo Z, Michael T, Grossman D, Gravano L, Zhai C, Herzog O, Evans DA Simple bm25 extension to multiple weighted fields.

[3] Kim J, Xue X, Croft WB A probabilistic retrieval model for semistructured data.

[4] Kim JY, Croft WB A field relevance model for structured document retrieval.

[5] Broschart A, Schenkel R Proximity-aware scoring for XML retrieval.

[6] Schlieder T, Meuss H (2002). Querying and ranking XML documents. *Journal of the American Society for Information Science and Technology*.

[7] Ogilvie P, Callan J Language models and structured document retrieval.

[8] Kamps J, De Rijke M, Sigurbjörnsson B (2005). The importance of length normalization for XML retrieval. *Information Retrieval*.

[9] Theobald A, Weikum G The index-based xxl search engine for querying xml data with relevance ranking.

[10] Theobald M, Bast H, Majumdar D, Schenkel R, Weikum G (2008). TopX: efficient and versatile top-k query processing for semistructured data. *VLDB Journal*.

[11] Gery M, Largeron C, Thollard F Ujm at inex 2008: pre impacting of tags weights.

[12] Trotman A, Lalmas M Why structural hints in queires do not help XML-retrieval.

[13] Geva S Gpx—gardens point xml information retrieval at inex 2006.

[14] Ogilvie P, Callan J Parameter estimation for a simple hierarchical generative model for xml retrieval.

[15] Mass Y, Mandelbrod M Using the inex environment as a test bed for various user models for xml retrieval.

[16] Wichaiwong T, Jaruskulchai C Mexir at inex-2011.

[17] Theobald M, Wang Q, Ramrez G, Marx MM, Theobald M, Kamps J Overview of the inex 2011 data-centric track.

[18] Ayala DV, Pinto D, Silverio SL, Castillo E, Vidal MT Buap: a recursive approach to the data-centric track of inex 2011.

[19] Wang Q, Gan Y, Sun Y Ruc at inex 2011 data-centric track.

[20] Schuth A, Marx M University of amsterdam data centric ad hoc and faceted search runs.

[21] Ramrez G Upf at inex 2011: books and social search track and data-centric track.

[22] Laitang C, Pinel-Sauvagnat K, Boughanem M Edit distance for xml information retrieval: some experiments on the datacentric track of inex 2011.

[23] Trotman A, Lalmas M The interpretation of cas.

[24] Trotman A, Sigurbjörnsson B Nex, now and next.

[25] Trotman A, Sigurbjörnsson B Narrowed extended xpath i (nexi).

[26] Robertson S, Zaragoza H (2009). The probabilistic relevance framework: BM25 and beyond. *Foundations and Trends in Information Retrieval*.

[27] Stephen R, Walker S, Jones S, Hancock-Beaulieu M, Gatford M (1994). Okapi at trec-3. *Overview of the Third Text Retrieval Conference*.

[28] Itakura KY, Clarke CLA A framework for BM25F-based XML retrieval.

[29] Aksyono A (2011). *Introduction to Search with Sphinx*.

[30] Wichaiwong T, Juruskulchai C (2012). A score sharing method for xml element retrieval information. *An International Interdisciplinary Journal*.

[31] Baeza-Yates RA, Ribeiro-Neto B (2011). *Modern Information Retrieval, The Concepts and Technology Behind Search*.

[32] Kamps J, Pehcevski J, Kazai G, Lalmas M, Robertson S Inex 2007 evaluation measures.

[33] Wichaiwong T, Jaruskulchai C (2011). Mexir: an implementation of high performance and high precision on xml retrieval. *Computer Technology and Application*.

[34] Wichaiwong T, Jaruskulchai C XML retrieval more efficient using ADXPI indexing scheme.

